# Understanding knowledge, attitudes and practices on Ebola Virus Disease: a multi-site mixed methods survey on preparedness in Rwanda

**DOI:** 10.1186/s12889-023-17251-w

**Published:** 2023-12-05

**Authors:** Janvier Karuhije, Menelas Nkeshimana, Fathiah Zakham, Benjamin Hewins, Justin Rutayisire, Gustavo Sganzerla Martinez, David Kelvin, Pacifique Ndishimye

**Affiliations:** 1 Rwanda Biomedical Centre, Ministry of Health, Kigali, Rwanda; 2 University Teaching Hospital of Kigali, Kigali, Rwanda; 3 Department of Virology, Faculty of Medicine, University of Helsinki, Helsinki, Finland; 4 Laboratory of Emerging Infectious Diseases, Department of Microbiology and Immunology, Faculty of Medicine, Dalhousie University, Halifax, Canada; 5 Izaak Walton Killam (IWK) Health Center, Canadian Centre for Vaccinology (CCfV), Halifax, NS Canada; 6 United Nations Children’s Fund (UNICEF), Kigali, Rwanda; 7 African Institute for Mathematical Sciences, Kigali, Rwanda

**Keywords:** Ebola virus disease, Qualitative research, Focus group discussion, Cross sectional study, Rwanda

## Abstract

**Background:**

The overall goal of this survey was to understand the knowledge, attitudes, and practices related to the Ebola Virus Disease (EVD) in Rwanda.

**Methods:**

This mixed-method cross-sectional survey was conducted in five selected districts of Rwanda. Quantitative data were collected from 1,010 participants using Kobo Collect Software and the analysis was performed using SPSS and Python software. Qualitative data were specifically collected from 98 participants through Key Informant Interviews (KIIs) and Focus Group Discussion (FGDs). Interview transcripts were imported into NVIVO 8 for coding and subsequent analysis.

**Results:**

As per our quantitative findings, we report that from the 1,010 respondents, 99.6% reported having previously heard of Ebola, 97.2% believed that vaccination is important in combatting the disease and 93.3% of individuals reported a willingness to receive vaccination should one become available. Around 54% of the respondents were correct in identifying that the disease is of a viral origin which originates from wild animals (42.1%). When asked if they believed that Rwanda is at risk of an EVD outbreak, 90% of the respondents believe that the country is at risk of an EVD outbreak, and the cofactors *gender* and *whether people dwell in Rubavu/Rusizi* were found to significantly impact their perception of threat. As per our qualitative findings, the respondents mentioned that both geographical proximity and relations with the Democratic Republic of Congo place Rwanda at risk of developing an internal outbreak. Although the respondents seemed to be aware of the Ebola prevention behaviours, it was noted that some of them will require significant time before reintegrating into the community an EVD survivor, as they will first need assurance that the patient has fully recovered. Therefore, the qualitative findings reinforce what we originally reported in the quantitative approach to this study.

**Conclusion:**

Our results show that there was high EVD-related knowledge and awareness among the general population in Rwanda. However, for strong public health awareness, preparedness, and protection, a massive investment should always be made in education about EVD with a special focus on districts neighboring countries where the disease is consistently being reported.

**Supplementary Information:**

The online version contains supplementary material available at 10.1186/s12889-023-17251-w.

## Background

Ebola Virus Disease (EVD), formerly known as Ebola hemorrhagic fever, is a severe and often fatal illness that affects humans. It was first discovered in 1976 near the Ebola River in what is now the Democratic Republic of the Congo (DRC) [1]. The average EVD case fatality rate is around 50% and the case fatality rates have varied from 40 to 90% in past outbreaks [2]. Originally transmitted to humans from wild animals, the spread of EVD is driven by human-to-human transmissions [3]. Consequently, community engagement is pivotal in the fight against EVD and successfully containing and controlling outbreaks. For effective outbreak control, the provision of comprehensive interventions including case management; infection prevention and control practices; surveillance and contact tracing; reliable laboratory services; safe and dignified burials and social mobilization are required [4, 5]. According to the World Health Organization (WHO), EVD vaccines have been used to help control the spread of Ebola outbreaks in Guinea and in the DRC [6]. There are currently two treatments approved by the U.S. Food and Drug Administration (FDA) to treat EVD caused by the Ebola virus, species *Zaire ebolavirus*, in adults and children. The first drug approved in October 2020, Inmazeb™, is a ‘cocktail’ combination of three monoclonal antibodies. The second drug, Ebanga™, is a single monoclonal antibody and was approved in December 2020 [7, 8].

The 2014–2016 West African EVD outbreak was the longest, deadliest, and most complex outbreak since the discovery of the virus in 1976 [5, 9]. It was not until March 29, 2016, that the WHO lifted the Public Health Emergency of International Concern status of this outbreak. With an estimated total cost of 4.3 billion USD, this epidemic resulted in 11,310 deaths and 28,616 laboratory confirmed cases. This was reflected by the substantial decrease in economic output in the three affected countries [2, 10, 11]. Guinea subsequently declared a new Ebola outbreak in February 2021, necessitating the establishment of preventative measures and mandates among its neighboring countries [12].

Since August 2018, the DRC has been facing a large-scale EVD outbreak in the eastern provinces of North Kivu and Ituri spanning as far as Goma and South Kivu provinces [13]. The DRC announced its tenth outbreak on August 1, 2018. Between April 30, 2018, and November 17, 2019, 3296 cases and 2196 mortalities were reported among the five most affected areas, those being (1) Benin with 697 cases, (2) Katwa with 674, (3) Mabalako with 416, (4) Butembo with 288, and (5) Mandima with 344. This outbreak has become the second largest EVD outbreak documented followed only by the 2014–2016 West African outbreak [14]. The unprecedented growth in cases (particularly in children) prompted the WHO to declare (July 2019) the Ebola outbreak in DRC a Public Health Emergency of International Concern [15, 16]. From 1 June 2020 to 18 November 2020, a total of 130 EVD cases including 119 confirmed and 11 probable cases were reported in the country [17]. In February 2021, a new EVD case was detected in Butembo, a city in DRC’s North Kivu Province [18]. Unstable conditions due to armed conflict [19], outbreaks of violence, and social/economic problems in affected areas complicated the public health response and increased the risk of disease spread both locally within the DRC and to other countries in the region including Uganda, Rwanda, Burundi, Zambia, South Sudan, and Central African Republic [20].

On 20 September 2022, Uganda declared an EVD outbreak caused by the Sudan Ebolavirus (SUDV) species, after the confirmation of a case in Mubende district in the central part of the country. During this outbreak, a total of 164 cases (142 confirmed, 22 probable), 77 deaths (55 among confirmed cases and 22 among probable cases) and 87 recovered patients were reported from September 20 to January 10, 2023 [21]. To date, Uganda has reported four SUDV outbreaks in 2000, 2011, two in 2012, and one in 2022 [22, 23]. The increased frequency of EVD outbreaks in DRC, Guinea and Uganda might be attributed to increased human-wildlife contact caused by extensive deforestation, hunting, and mining practices (among other reasons), which demonstrates a need for a robust monitoring system to inform future preventative policy measures [14, 24].

Rwanda’s dense population and frequented public transportation system render it highly susceptible to the rapid spread of EVD. Sharing borders with the DRC and Uganda increases Rwanda’s risk of cross border EVD transmission due to the unmonitored cross-border movement of people and goods and weak border control systems. The increase in magnitude of the 2019 DRC EVD outbreak in Goma (in July 2019) and the Mwenge Health Zone in South Kivu (in August 2019) further increased the risk of cross border EVD transmissions in Rwanda. Borders between Rwanda, DRC, and Uganda are particularly vulnerable as they are only separated by border check points. These borders, especially those shared with DRC, are characterised by intense trade activities and a high volume of population movement/migration. The threat of cross-border EVD transmissions is an emerging and increasing cause for concern not only for Rwanda but Burundi as well. Rwanda’s constant vulnerability to cross border EVD transmission necessitates strengthened EVD preventative and preparedness interventions, greater cross-border collaboration, and the flexibility to adapt to emerging needs [25].

The Government of Rwanda, through the Rwanda Biomedical Centre, launched the Multi-sectorial Risk Communication and Community Engagement Strategy for Ebola Virus Disease Preparedness and Response in August 2018 [25]. The strategy seeks to contribute to the national preparedness and response plan to halt EVD transmission in Rwanda, through effective evidence based social mobilization, community engagement, and public education that advocates for socio-behavioural changes. Although Rwanda remains free of Ebola, its cost-effective control strategy requires an up-to-date understanding of peoples’ knowledge, beliefs, attitudes and behavioural patterns surrounding the virus. Apart from unpublished descriptive technical reports, this survey is the first to assess EVD-related knowledge, attitudes, and practices at individual, institutional, and societal levels in Rwanda.

## Methodology

### Study design

**Fig. 1:**
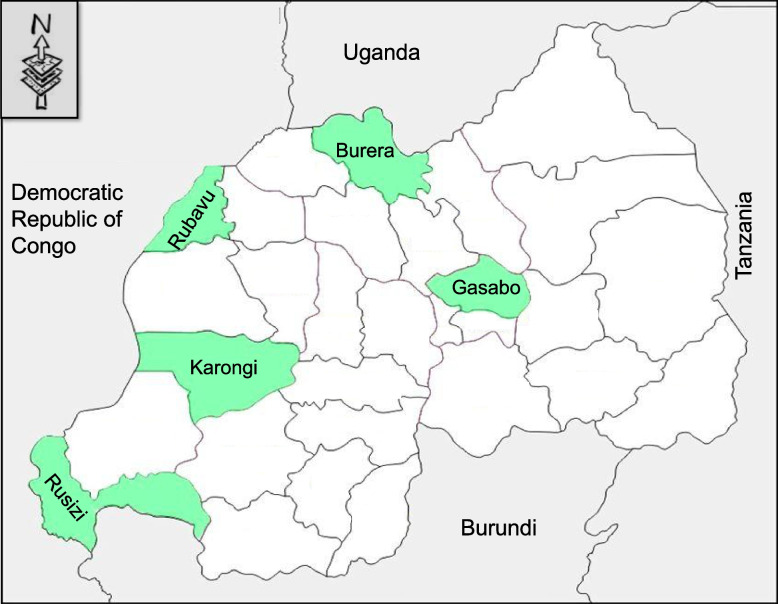
Map of Rwanda highlighting survey sites (green). This map is a modified version from the map originally obtained at https://d-maps.com/

This mixed-method cross-sectional survey was conducted from May to July 2021 in five selected districts from Rwanda (Fig. 1). These districts were purposely selected from the districts identified as high-risk zones given their geographical proximity (Rusizi, Karongi, Rubavu and Burera) or with direct air links (Gasabo) to DRC and Uganda.

### Quantitative approach

This research was carried out in the initial phase of the COVID-19 pandemic, and due to the Government of Rwanda’s COVID-19 travel restrictions, all interviews were conducted over the telephone by a trained member of the research team. The research team consisted of two groups. The first group was composed of data collectors who were stationed in all five study districts. Their role was to sample the study participants, obtain informed consent, and collect participants’ contact information. The second group consisted of enumerators trained to administer the questionnaire. Later, the enumerators called the participants to conduct the interviews. Participants were randomly selected from urban and rural sectors in each village. The study participants were parents/caregivers or household members (male and female) of various backgrounds and occupations. One participant was randomly selected from each household selected for the study. Vulnerable groups such as child headed families/households (where there are no adult caregivers and children live on their own), people living with disabilities, and others were deliberately selected and added to the study cohort. Participants who could not communicate, below 18 years old, were excluded from the study. The sampling frame in each village was developed with the help of community leaders. Assuming 50% of the target population have an attribute of interest, a minimum sample of 1000 was found to be adequate at 95% confidence level, 4.95% level of precision and considering a non-response rate of 15% [26]. In all five districts, the research team contacted a total of 1010 participants using a structured questionnaire: Gasabo (205), Karongi (203), Rubavu (207), Rusizi (193), and Burera (202).

The questionnaire was originally designed in Kinyarwanda and was later translated to English by two bilingual experts, before the study team members validated the questionnaire. In order to assess the convenience and interpretation of the questionnaire, a pilot study was carried out on 35 participants from the general population and the questionnaire was modified accordingly. The questionnaire was predominantly comprised of: *i*) demographical questions (i.e., age, gender, marital status, place of residence, religion; education level, and occupation); *ii*) EVD knowledge questions (i.e., have you heard of EVD before; what is EVD; what are the symptoms; what causes EVD; how do people contract EVD; how can people prevent contracting EVD; have you received education and training relating to EVD; who are the trusted community leaders; have you heard of EVD in the neighbouring countries; do you think Rwanda is at risk; what source of information do you have on EVD; what is the most trusted source of information; what is your preferred method of being informed/receiving information; and are you personally at risk); *iii*) EVD contact and treatment (i.e., do you think traditional/spiritual healers can treat EVD; would you take your relatives to a treatment centre; what would you do if a family member contracted EVD; what actions would you/your family implement to avoid contracting EVD; have you changed your behaviour to avoid being infected; what would you do if a person died of EVD; if your family member died of EVD, what would you do; have you been involved in prevention activities; if a person had EVD, would you touch them; if a person had EVD, would you shake hands with them; if a person had EVD, would you work or study with them; if a person had EVD, would you buy food from them; if a person had EVD, would you have sex with them); *iv*) prevention measures (i.e., what recommendations can you give to the ministry of health; do you believe that vaccination is important for preventing EVD infection; do you believe that EVD vaccines are safe; what factors do you believe would prevent people in your community from receiving an EVD vaccine, if one was available).

### Qualitative approach

A purposive sampling strategy was used to select KIIs and FGDs participants from the list of contacts provided by the district-based data collectors. A total of 98 people participated through KIIs and FGDs. The reasons for selecting these participants were mainly to ensure diversity in our cohort (demographics and backgrounds), availability and accessibility of participants, and willingness to openly express their opinions and experiences. In each district, KIIs were completed with teachers, Early Childhood Development (ECD) care givers, Community Health Workers (CHWs) and Health workers in health centres. At the national level, KII participants were representatives from Government institutions and their key collaborators. All interviews were conducted over the telephone, or via the Zoom platform. In each district FGDs were conducted with community leaders, parents/caregivers, and adolescents. Each FGD consisted of four people and were conducted through conference calls. For the quantitative study, the KIIs and FGDs guides were composed of questions related to knowledge and awareness surrounding the cause(s) of EVD, signs and symptoms; Risk perceptions and beliefs; Behaviours’ and practices; Information communication channels and sources’; Recommendations and vaccination Issues.

### Data analysis

Quantitative data were collected using Kobo Collect Software and then converted to an excel sheet. Analysis was carried out using Statistical Package for Social Sciences (SPSS) version 20 (Chicago, IL, USA) and Python version 3.9.7. Chi-squared tests were used to determine the association between survey responses and district, marital status, and education level of the respondents. A logistic regression model was used to assess the relationship between the question “Do you think Rwanda is at risk of EBV an outbreak?” and the cofounder factors age, gender at birth, district, level of education, and marital status. The minimum *p* value for significance was considered 0.05.

Qualitative data was managed using NVIVO 8 software. Interview transcripts were imported into NVIVO 8 for coding and subsequent analysis [27]. To ensure inter-coder reliability and agreement, coding was performed by two researchers. Inter-coder agreement remained above 90% throughout the analysis.

## Results

### Quantitative findings

#### Characteristics of the study population

Out of 1010 respondents surveyed across the five districts, 20.5% were in Rubavu, 20.3% in Gasabo, 20.01% in Karongi, 20.0% in Burera and 19% in Rusizi. In all surveyed districts, 56% of respondents were male, ranging from 49.2% (Rusizi), 52.7% (Gasabo), 57.9% (Burera), 59.1% (Karongi) to 60.4% (Rubavu). For the total sample, approximately 70.3% of participants were married, 25.3% were never married, and the most respondents (50%) reported that they had obtained a primary level of education (Table.1).

**Table 1:** Characteristics of the study population, *n* = 1,010

Variable	Category	Gasabo	Karongi	Rubavu	Rusizi	Burera	Overall
n (%)		205 (20.3)	203 (20.1)	207 (20.5)	193 (19.1)	202 (20.0)	1010
Sex (%)	Male	52.7	59.1	60.4	49.2	57.9	56
	Female	47.3	40.9	39.6	50.8	42.1	44
Education (%)	No formal education	14.1	6.4	8.7	4.1	2.5	7.2
	Primary	50.2	49.3	36.7	56.5	57.9	50.0
	Secondary	27.3	32.5	42	33.7	32.7	33.7
	Tertiary	1.5	1.0	2.9	1.0	1.5	1.6
	University	6.8	10.8	9.7	4.7	5.4	7.5
Marital Status (%)	Single	29.3	11.8	29	32.6	24.3	25.3
	Married	63.9	85.2	67.6	62.7	71.8	70.3
	Widowed	3.4	3.0	1.4	4.1	3.5	3.1
	Divorced	3.4	0	1.9	0.5	0.5	1.3

#### Assessment of knowledge and awareness on Ebola

The overall Ebola knowledge levels disaggregated by district are shown in Table 2. The analysis of these data suggests relatively high levels of awareness and knowledge pertaining to Ebola across the five study districts albeit with variations in geographical proximity to affected regions of DRC. The study participants responded that they receive Ebola-related awareness and knowledge from multiple sources, some of which (media sources) are prevalent across the population sub-groups. The results are confirmed by the low *p* values obtained in a chi-squared test comparing answers from different districts, which indicates heterogenous answers across different places of the country. For instance, when people were asked if EVD is caused by a virus the highest proportion of correct answers was found in Rubavu (which is a main border to the DRC) whilst the lowest was found in Burera (which does not border the DRC). This difference is computed by a low *p* value (i.e., < 0.001).

**Table 2:** Knowledge and awareness on Ebola

	**Gasabo**	**Karongi**	**Rubavu**	**Rusizi**	**Burera**	**Overall**	***p* value (chi-squared test)**
**Have you ever heard of Ebola**							
Yes	100.0	99.5	100.0	98.4	100.0	99.6	0.005
No	0.0	0.5	0.0	1.6	0.0	0.4	
**Perceived Causes of the Ebola Virus**							
Virus	58.0	42.4	58.5	83.4	30.2	54.3	< 0.001
Bats / monkeys / Chimps	32.2	62.1	37.7	30.1	48.0	42.1	< 0.001
Other	6.4	15.3	2.4	2.0	15.8	8.5	-
**Transmission ways**							
Airborne^a^	42.0	44.8	15.5	52.3	27.7	36.2	< 0.001
Preparing bushmeat/Eating bush meat	34.1	42.4	38.6	22.8	33.7	34.5	< 0.001
Bodily fluids of infected persons	72.2	53.7	79.2	48.7	51.0	61.2	< 0.001
Mosquito Bite^a^	7.3	1.0	2.4	2.1	4.0	3.4	0.004
Shaking hands with infected persons	40.5	39.9	74.4	30.1	45.5	48.3	< 0.001
Using utensils used by infected persons	5.4	36.0	32.4	25.9	22.3	24.4	< 0.001
Other	0.5	5.4	2.4	0.0	19.3	5.6	< 0.001
Don’t Know	7.3	3,9	2.9	4.1	9.4	5.5	0.02
**Signs and Symptoms**							
Fever	81.5	80.3	87.0	83.9	91.1	84.8	< 0.001
Headache	42.9	27.1	57.0	29.5	32.7	38.0	< 0.001
Muscle pain	19,0	10.3	30.4	9.8	20.8	18.2	< 0.001
Diarrhoea	34,1	43.3	47.3	33.7	33.7	38.5	< 0.001
Abdominal pains	15.6	3.0	25.6	9.8	11.9	13.3	< 0.001
Sore Throat	9.3	3.9	23.7	6.2	5.0	9.7	< 0.001
Vomiting	32.7	45.3	40.6	44.0	38.6	40.2	0.007
Bleeding	78.5	76.8	94.2	54.1	93.6	80.7	< 0.001
Fatigue	38.5	9.9	4.8	39.4	21.8	31.7	< 0.001
Other	3.9	1.9	1.9	1.0	5.9	3.5	< 0.001
**Prevention measures**							
Avoid contact with infected persons	95.1	95.6	92.8	93.8	84.7	92.4	< 0.001
Not eating uncooked bush meat	36.1	41.9	34.8	19.2	29.7	32.5	< 0.001
Bathing with salt and hot water^a^	25.4	6.4	31.4	5.2	17.8	17.4	< 0.001
By avoiding mosquito bites^a^	4.4	0.0	10.1	4.1	3.0	4.4	< 0.001
Not participating in burial rituals	11.7	26.6	30.4	22.8	22.8	22.9	< 0.001
Not attending funerals	2.4	16.7	18.8	4.1	22.3	13.0	< 0.001
Other	4.4	10.8	10.6	1.0	30.2	11.5	< 0.001
**Received any training or health education on Ebola**							
Yes	11.7	19.7	15.9	25.9	26.2	19.8	< 0.001
No	88.3	80.3	84.1	74.1	73.8	80.2	
**Source of Information on Ebola**							
Radio	95.1	93.1	92.2	96.8	92.6	93.9	0.28
TV	60.3	17.7	51.9	35.5	6.9	34.6	< 0.001
Social Media	23.0	16.3	29.1	17.7	6.9	18.7	< 0.001
Mobile Phone text messages	12.3	15.8	11.2	24.2	4.0	13.3	< 0.001
Health workers	18.6	23.2	18.4	31.7	23.3	22.9	0.01
Flyers/Posters	12.7	2.0	17.5	16.7	11.9	12.1	
Church/Religious workers	6.4	17.2	5.8	7.0	10.4	9.4	< 0.001
Traditional healers	0.5	0.0	1.0	0.0	0.0	0.3	0.01
Email	0.0	1.0	1.5	2.7	0.0	1.0	< 0.001
Drama	4.4	2.0	0.0	0.0	0.5	1.4	< 0.001
Newspapers	11.3	1.0	4.4	16.7	4.0	7.3	< 0.001
CHW	9.8	23.6	20.4	22.0	26.2	20.4	0.27
Other	1.0	2.5	4.9	1.1	21.8	6.3	0.04
**Trusted Sources of Information**							
Health professionals	71.1	56.7	68.4	69.1	60.4	65.1	-
Community health workers	23.0	33.5	28.6	22.3	26.2	26.8	-
NGO	2.5	0.5	0.0	4.3	0.0	1.4	-
Traditional leaders	0.0	0.0	0.0	0.5	0.0	0.1	-
Religious leader	0.0	1.0	0.0	1.6	0.5	0.6	-
Other	3.4	8.4	2.9	2.1	12.9	6.0	-

^a^Wrong response

#### Assessment of attitudes towards Ebola infected and affected persons

Overall, the respondents in all districts showed a positive attitude towards EVD survivors. For instance, 88% of surveyed respondents (Table 3) reported that they would welcome back a neighbor who survived Ebola, with the highest proportion being in Karongi (95%) and the lowest in Burera (82%), the heterogeneity in the answer is captured by a low *p* value (i.e., < 0.001) in the chi-squared test. However, the proportion of respondents who would hug or touch a person who was previously infected with EVD was lower (i.e., 72.3%, in general) with the highest proportion to answer yes being found in Karongi (93.6) and the lowest in Rusizi (61.9%); this discrepancy in answers is reflected by a low *p* value (i.e., chi-squared test < 0.001).

**Table 3:** Knowledge attitudes towards Ebola infected and affected persons

	**Gasabo**	**Karongi**	**Rubavu**	**Rusizi**	**Burera**	**Overall**	***P* value (chi-squared test)**
**Proportion of respondents who would welcome back an EVD survivor**							
Yes	91.2	95.1	86.5	84.5	82.7	88.0	< 0.001
No	6.3	1.5	6.8	9.8	14.4	7.7	
Not sure	2.4	3.4	6.8	5.7	3.0	4.3	
**Proportion of respondents who would touch or hug an EVD survivor**							
Yes	81.0	93.6	64.7	59.6	61.9	72.3	< 0.001
No	14.6	3.4	32.9	29.5	33.7	22.8	
Not sure	4.4	3.0	2.4	10.9	4.5	5.0	

#### Assessing the risk perceptions and beliefs about Ebola

Table 4 shows the respondent risk perceptions and beliefs related to EVD. First, we report the DRC to be the country with the highest proportion (overall 99.7%) of risk perception associated. The high *p* value indicates homogenous answers across districts. Moreover, the highest proportion of respondents (90%) believe that Rwanda was at risk of an Ebola outbreak and 87.8% of the participants think that, individually, they are at risk. However, regarding individual beliefs of whether Rwanda was at risk of an EVD outbreak, we found low *p* values using a chi-squared test controlling for districts, indicating heterogeneity in responses. In terms of the question “are you personally at risk”, we found low *p* values comparing districts, meaning heterogeneity in the answers, with the lowest proportion being in Rusizi (66% yes and 33% no) and the highest in Karongi (86% yes and 14% no). Finally, most of the respondents do not believe in traditional and spiritual-based therapies for treating EVD (91.5% and 89.6%, respectively), with heterogeneous responses across districts, i.e., the highest proportion of “no” in Rusizi (83.5 and 79.5, respectively) and the highest in Rubavu (95.2% and 95.2%, respectively).

**Table 4:** Knowledge the risk perceptions and beliefs about Ebola

	**Gasabo**	**Karongi**	**Rubavu**	**Rusizi**	**Burera**	**Overall**	***p*** **value (chi- squared test)**
**Respondent perception of risk associated with the countries affected by Ebola by study districts**							
DRC	99.4	99.0	100.0	100.0	100.0	99.7	0.28
Uganda	8.8	5.7	2.1	1.3	5.8	4.7	0.008
Burundi	1.3	6.7	0	0	0	1.7	< 0.001
Other	3.8	1.0	0.5	1.9	0	1.3	0.026
**Do you think Rwanda is at risk of Ebola outbreak**							
Yes	89.5	92.0	93.7	79.4	93.9	90.0	< 0.001
No	10.5	8.0	6.3	20.6	6.1	10.0	
**Are you personally at risk of getting Ebola**							
Yes	78.0	95.6	91.3	80.3	93.6	87.8	< 0.001
No	22.0	4.4	8.7	19.7	6.4	12.2	
**Level of risk to getting infected with Ebola**							
Very high	8.8	6.9	10.2	2.5	31.1	11.9	-
High	22.1	17.3	36.4	30.7	12.8	21.3	-
Moderate	39.7	33.2	25.7	30.7	17.2	29.6	-
Low/Minimal	29.4	42.6	27.8	49.7	38.9	37.3	-
**Do you believe that traditional healers can treat Ebola**							
Yes	3.4	2.5	1.9	1.6	2.5	2.4	< 0.001
No	88.8	95.6	95.2	83.5	94.1	91.5	
Not Sure	7.8	2	2.9	14.9	3.5	6.1	
**Do you believe that spiritual healers can treat Ebola**							
Yes	5.4	4.9	1.0	3.7	4.5	3.9	< 0.001
No	85.9	93.1	95.2	79.5	93.6	89.6	
Not Sure	8.8	2.0	3.9	16.8	2.0	6.6	

#### Assessment of Ebola related behaviours and practices

The respondents were also asked to indicate the action they would take for themselves and family members to avoid Ebola. Table 5 presents the proportion of respondents with respect to various personal hygiene practices to avoid Ebola. The most common preventive measures, which were mentioned in each of the locations, were hand washing and limiting body contacts. We also found that practicing handwashing and good personal hygiene was a common behavior to avoid Ebola across districts, with little variation (*p* = 0.144), while heterogeneously (*p* < 0.001), people reported changing their behavior or practices to avoid being infected with Ebola, with the lowest proportion of “yes” answers found in Gasabo (60.5%) and the highest in Rubavu (91.8%). When asked if people believe it is important to receive vaccination to prevent Ebola, 97.2% answered “yes” with non-significant variations across the districts (*p* = 0.23). However, people from different districts showed variability in their answers regarding whether they believe vaccines against EVD are safe. Overall, 90.1% of participants answered “yes”, while the lowest proportion of “yes” answers were found in Rusizi (86%) and the highest in Gasabo (97.1%), the heterogeneity in the answers reflects a low *p* value for this question (i.e., < 0.001).

**Table 5:** Knowledge of Ebola related behaviours and practices

	**Gasabo**	**Karongi**	**Rubavu**	**Rusizi**	**Burera**	**Overall**	***P*** **value (chi-squared test)**
**Personal Behaviours / hygiene practices to avoid Ebola**							
Practice handwashing and good personal hygiene	86.8	87.7	91.8	83.4	85.6	87.1	0.144
Avoiding touching sick people or the dead	75.1	65.0	65.7	81.3	54.5	68.2	< 0.001
Not eating raw bush meat	15.1	28.6	15.9	17.6	23.8	20.2	< 0.001
Washing with salt and hot water	20.5	0.0	14.5	2.6	5.4	8.7	< 0.001
Other	2.9	7.9	8.2	0.5	19.3	7.8	< 0.001
**Have you changed your behaviour or practices to avoid being infected with Ebola?**							
Yes	60.5	79.8	91.8	69.9	65.3	73.6	< 0.001
No	34.6	4.4	6.8	15.0	33.7	18.9	
Not sure	4.9	15.8	1.4	15.0	1.0	7.5	
**Behaviours in the event a family member contracted Ebola**							
Do nothing	0.5	0.0	0.0	0.0	0.0	0.1	-
Hide the sick person	1.0	0.0	0.0	0.0	0.0	0.2	-
Isolate the sick person	13.7	3.0	21.3	5.3	5.9	9.9	-
Call the hotline	53.7	68.0	54.1	28.6	61.4	53.5	-
Take patient to health centre or hospital	21.0	16.3	18.8	38.6	13.9	21.5	-
Take patient to an Ebola Treatment Centre	9.8	2.5	3.9	27.5	2.0	8.8	-
Other	0.5	10.2	1.9	0.0	16.8	6.0	-
**Do you believe that it is important to get vaccinated to prevent Ebola**							
Yes	97.6	96.6	99.0	95.3	97.5	97.2	0.23
No	2.4	3.4	1.0	4.7	2.5	2.8	
**Do you believe that vaccines against Ebola are safe**							
Yes	97.1	92.1	87.0	86.0	88.1	90.1	< 0.001
No	2.9	7.9	13.0	14.0	11.9	9.9	

#### General perception

To assess the impact of the variables age, gender, district of residence, education level, and marital status in people answering the question “Do you think Rwanda is at risk of an EVD outbreak?”, we performed a logistic regression analysis (Table 6). We report significant effects on an answer for the question (i.e., either yes or no) for the gender of the respondent and whether the respondent dwells in Rubavu or Rusizi.

**Table 6:** Logistic regression analysis

		Do you think Rwanda is at risk of EVD outbreak?
		*p* value
Age		0.853
Gender		0.038
District	Gasabo	0.783
	Karongi	0.307
	Rubavu	0.048
	Rusizi	< 0.001
	Burera	0.046
Level of education	None	0.123
	Primary	0.429
	Secondary	0.423
	Tertiary	0.989
	University	0.307
Marital status	Single	0.564
	Married	0.804
	Widowed	0.997
	Divorced	0.989

### Qualitative findings

#### Assessment of knowledge and awareness on Ebola

Analysis of qualitative data suggests that there are variations on overall Ebola-related knowledge among the sub-population groups: Health Workers, Community Leaders, Adolescents and Teachers for both ECD and Secondary schools.

*We have learned that Ebola is caused by the virus which has its origin in animals* *:* *FGD, Community Leader Rubavu (Rural).*

*I do not know the causes of Ebola, but I know its symptoms like vomiting, bleeding and strong fever, things like that. KII, Teacher Karongi (Urban)*.

*I wish I had knowledge on Ebola. I could explain to students, I do not have enough knowledge. I am not ignorant completely. I know little about it such as what we hear from radios on the nature of the epidemic. Teacher Karongi (Rural)*.

Although Rwanda has not yet recorded an EVD outbreak, the study respondents were knowledgeable regarding the prevention of future outbreaks, and the knowledge was almost uniform across the districts, study population sub-groups, and geographical areas.

*Yes, Ebola is a preventable disease. The first prevention measure is cleaning, disinfection or disposal of items that may have been contaminated by body fluids. KII, Teacher, Karongi (Rural)*.

*Prevention of Ebola is washing hands regularly, avoiding crowded places or places where there is an outbreak of Ebola. FGD, Parent Caregiver, Burera (Rural)*.

*We can prevent Ebola through different measures such as washing hands, avoiding assembling, avoiding shaking hands and more important getting vaccinated against Ebola*. *FGD, Adolescent, Rubavu (Urban)*.

Overall, the respondents reported receiving knowledge on Ebola mainly from their community leaders and community health workers.

*During community work called “umuganda” health workers are invited to share information on Ebola. However, since March 2020, there has not been any such educative talks on Ebola. FGD, Parent/Caregiver, Rusizi (Rural)*.

*In Rusizi, there is a programme of vaccination against Ebola, so community leaders have information on Ebola. Also doctors from Kigali help people in the district on how to take measures to prevent Ebola. FGD, Community Leader, Rusizi (Urban)*.

Several districts have been organizing EVD awareness campaigns to educate the public about how to better prevent the spread of the virus. For instance, community leaders in Rubavu indicated that they had received formal Ebola training from health workers at the district level as a part of the district awareness raising strategies.

*All Community leaders and opinion leaders have been trained and involved in Ebola education. The training was an alert in Rubavu district. We even did campaigns and the cars were driving around the town with loudspeakers with instructions on knowledge on Ebola and preventive measures. FGD, Community Leader, Rubavu (Urban)*.

The analysis of the feedback from participants identifying as teachers across the study districts noted little to no formal (or informal) Ebola-related dissemination of knowledge and/or training for teachers. For instance, teachers in Karongi reported limited exposure to educative activities on Ebola-related knowledge. One of the teachers had this to say:

*Honestly, I do not know much about Ebola. We simply just get some information from the radio, but this is not comprehensive for the role we play as teachers who should impart such knowledge to the children, who would in turn also share the information with their families at home. KII, Teacher, Karongi (Urban)*.

#### Assessment of attitudes towards Ebola infected and affected persons

Responses from the FGDs with parents and local leaders indicated that, in general, the community members have a positive attitude towards survivors. The following is an excerpt from one of the participants.

*Sometimes the person can be quarantined. However, when he or she is sent to the hospital and the hospital discharges him/her because he/she is cured, then there is no reason to fear or quarantine him/her. We can welcome him/her back*. *FGD, Parent/Caregiver, Karongi (Urban)*.

Conversely, the results also show that some respondents have negative attitudes towards Ebola survivors, should they affect the country. These results are also supported by information gathered through FGDs. For instance, some participants will need more time to personally assess the risk of eating, hugging, or shaking hands with a person who has recovered from Ebola.

*For the recovered member, it takes time to go back into the community, the population would like to be sure that he has fully recovered, but there is no discrimination. FGD, Local Leaders, Rubavu, (Rural)*.

Parents and local leaders also reported a willingness to adhere to Ebola burial guidelines and protocols when burying someone they suspect that he has died of Ebola (i.e., no touching the corpse and allowing the burial team to come and bury the body). The following are excerpts from the FGDs conducted with the parents.

*For someone who died of Ebola, there is a burial team and because of fear of Ebola no one can approach the dead body, the community accept use of burial team, even if families are psychologically affected by not paying the last respects to the dead*. *FGD, Parents/Caregivers, Gasabo (Urban)*.

*In our customs we are used to make the last respect to the dead person, so it is too hard to accept the burial team, but it is a must, sometimes one member of the family can wear the protective equipment and be part of the burial team. FGD, Local leaders, Rubavu (Rural)*.

One National level KII noted the need to conduct a refresher on EVD to raise awareness.

*My immediate suggestion would be to conduct a refresher related to Ebola especially when it comes to community sensitization approaches, latest information, latest science about Ebola, strengthen the knowledge of community health workers about the vaccine. Also because of turnover, this refresher will be new information for new health workers. We should also have a branded corner in health facilities where people can access Ebola information. We also need to engage teachers, ECD caretakers, young children to institutionalize knowledge about Ebola, plus we should constantly update to highlight the difference between COVID-19 and Ebola. We also need to have a refresher for religious leaders. KII, UNICEF, Gasabo (National).*

#### Assessing the risk perceptions and beliefs about Ebola

Most of the respondents think that Rwanda was at risk of an Ebola outbreak. This is reflected in the FDG/KII findings with respondents citing both DRC and Uganda as the source of regional Ebola cases.

*“Yes, the epidemic can come in Rwanda because our neighbour DRC is regularly suffering from the epidemics and we are at a risk since they can happen to a person from DRC who can for instance enter and contaminate or spread the Ebola”, KII, Teacher, Gasabo, (Urban)*.

The fact that neighbouring countries such as DRC and Uganda had reported Ebola cases put the Rwandans at high risk. The following excerpts suggest the potential risk of Ebola as a result of illegal movement between boarders.

*Where we live, there are people who come from DRC to pick sand. We need to avoid contact with these people. They are picking sand at a place called Kiraro. FGD, Adolescent, Karongi (Urban)*.

*With new DRC, one method of transport is boat. Many people come from Goma and Bukavu and there it is possible for Rwandans to get Ebola. Also, here in Karongi, fishing is an important business. The lake is between two countries, but borders are not clear and there is constant movement between these people doing this business and other businesses which make the spread of Ebola possible. FGD, Community Leader, Karongi (Urban)*.

“*It has been said Ebola is coming from bats and in Rwanda we have those bats. So, it can come also. In addition, movement from Goma to Rwanda can facilitate the spread of the virus*”. *FGD Adolescents, Gasabo (Urban)*.

Most of the respondents from the FGDs and KIIs perceive that everyone in Rwanda takes Ebola as a very serious disease that spreads. However, isolated responses from FGDs with adolescents in Burera, and parents in Gasabo, Karongi, Rubavu and Burera mentioned that Ebola was a political disease, one that is ‘made-up’ by politicians.

*“Many people in the community know the seriousness of Ebola. Before COVID-19 pandemic, Health workers from several hospitals were sent into different churches to teach them how to take measures against Ebola virus”. FGD, Community Leaders, Karongi (Rural)*.

*“Once I heard traders talking about a political disease”, FGD, Adolescent, Burera (Rural)*.

*“People have different way of understanding. Some people have the misconceptions on Ebola. Some said that Ebola is a political disease, it does not exist”. Parent FGD, Gasabo (Urban)*.

The majority of myths and misconceptions were cited in KIIs and FGDs conducted in Gasabo, and the majority were captured in the KIIs with health workers. The persistence of myths and misconceptions also indicates a need for EVD awareness campaigns.

*“The myths and misconception about EVD, it is said that the epidemic is caused by a virus made from laboratories and brought in Africa”. KII, Health Worker, Gasabo (Urban)*.

“*It is somehow said that it is an antichristian epidemic and aiming at imparting satanic stamp of 666*”. *KII, Health Worker, Gasabo (Urban)*.

#### Assessment of Ebola related behaviours and practices

Respondents were asked to indicate whether they had changed their behaviour or practices to avoid being infected with Ebola. This change in behaviour was attributed to the community meetings/umuganda that were held before the COVID-19 pandemic. At these meetings, the members of the community were trained and equipped with various methods to mitigate contracting the disease. These training sessions were performed by health workers who gave speeches and handed out materials at these events.

*During the community work also, we were given the opportunity to talk to the people. When you suspect a person, you mention it on his transfer then you wait for the answer from the health centre to see if you were right*. *KII, Health worker, Karongi (Urban)*.

In the event of onset of symptoms or confirmation of an Ebola case within a family, respondents across all the groups (FGDs & KIIs) cited isolation as the recommended practice. Interestingly, parents in Gasabo went on to mention that isolation should be followed with testing of all family members to determine whether the disease had spread within the family.

“*In my opinion, in case families suspect Ebola, we have to inform our community health workers in my village, because I know, they have more information on health issues. Usually, we always seek help from community health workers because they are close to us and know how to behave in case of illness, we trust them, but in the meantime, we have to isolate the suspect*”. *FGD, Parent/caregiver, Gasabo (Urban)*.

“*When we suspect one of member to be infected better to call the health workers while avoiding any contact and approach to that person*”. *FGD, Parent/caregiver Rusizi (Rural)*.

Some respondents indicated that they were previously vaccinated or willing to be vaccinated, highlighting the importance of EVD vaccines in preventing further infections and death. The vaccinated participants attested to experiencing no side-effects from taking the EVD vaccine.

*I saw many being injected especially those bordering Democratic Republic of Congo and I was wondering when we will get the same vaccines too, unfortunately we did not qualify for the first round, but many are ready for the EVD vaccines*. *FGD, Community Leader, Karongi (Rural)*.

*I have already been vaccinated and my family. If there are preventive measures, they are not 100 percent tight. The only way to be definite is to get vaccinated*. *FGD, Community Leader, Rubavu (Urban)*.

*My family has been vaccinated for Ebola. We did not experience any problem. We think the vaccine is safe because it is tested on those that received it.* FGD, Community Leader, Rubavu (Urban).

Most of the respondents were with the opinion that they saw no reason for communities to refuse vaccination. A few participants; however, cited myths and rumours (microchip or causing infertility) as the cause for hesitancy but also believed this could be overcome through awareness campaigns and community mobilisation.

*Nothing in my community can prevent people to get vaccinated, sometimes rumours but with sensitization it was over. FGD, Community leader, Rusizi (Rural)*.

The primary channel of communication that is most preferred by the respondents is radio, and in some cases, Television (TV), but radio stands out across key informants and focus groups.

*“The Radio is the principal means since almost everyone has it. You can invite people in the community for a meeting and few will come but when it comes to the announcement made on the radio, everyone will attend.” KII, Teacher, Karongi (Urban).*

*“Primarily they (communities) listen to the radio, they watch television and also listen to cars going around with big speakers.” KII, Health Worker Rubavu (Rural)*.

Pertaining to trusted sources of information, the respondents collectively viewed the Ministry of Health and its personnel, Government Institutions, such as Rwanda Biomedical Centre, and local leaders as being more trustworthy in times of disease outbreaks.

*“Those who do meetings like Country leaders at the country level. We cannot accept the information from the district unless we hear ourselves from high rank officials. Unless it comes from the cabinet, otherwise we can’t trust the rest.” KII, Teacher, Karongi (Rural)*.

Regarding accurate dissemination of information, some participants indicated that there is need to strengthen the flow of educational information regarding Ebola. One of the key informants said the following.

*What is important is to reinforce the flow of information so that there is no cut in the communication to ensure that the right information is in the right place at the right moment. we monitor this very closely also to make sure that there are no rumours. KII, WHO, Gasabo (National)*.

## Discussion

This study sought to assess the knowledge, attitudes and preventive practices concerning EVD in five selected districts at high risk of cross-border spread given their geographical proximity (Rusizi, Karongi, Rubavu and Burera) or with direct air links (Gasabo) to DRC and Uganda. We observed relatively high Ebola-related knowledge and awareness levels across the five study districts albeit with variations by geographical proximity to affected regions of DRC and by population subgroups. Such a high level of awareness is a good indication of the awareness raising efforts that have been previously undertaken by the Ministry of Health. Consultations with health workers across the five districts indicated that they had received formal training on Ebola prior to the COVID-19 pandemic, which had covered topics on general knowledge surrounding Ebola, such as what the causative agent is, how it is spread, how it can be prevented, as well as the hallmark signs and symptoms. The training is likely to have contributed to a relatively high level of knowledge on Ebola. Apart from this training for health workers, most respondents across the five districts reported that they mainly received the information on Ebola through media sources, including radio and TV. Another proportion of respondents reported receiving information on Ebola from posters/notice boards at health facilities. People who came from the DRC were also a source of knowledge for the adolescents. The channel of communication that is most preferred among respondents from all districts is radio, which may be attributed to its increased accessibility (when compared to TV or social media). This finding, while not surprising, underscores the importance of radio in the context of health-related communication, especially in rural areas where other media sources are less accessible.

To the best of our knowledge, this is the first comprehensive KAP survey on EVD in Rwanda. Our findings indicated a heightened level of Ebola risk perception in Rwanda as a country because it shares a border with DRC, which is the EVD epicentre in the East African region. Such a study is essential for developing preventive interventions for community members considering the increased risk of cross border EVD spread between both countries. Previous reports and studies have also showed that the risk of cross border EVD spread is exacerbated by the high volume of people traveling across the different borders between Rwanda, DRC and Uganda [25, 28].

Despite the generally high level of Ebola-related knowledge, gaps remain in respondents’ knowledge and attitudes towards EVD that were likely to impact preventive practices and behaviours. This was observed in survey results where a large proportion of people were apprehensive about whether they should physically and/or socially interact with EVD survivors. For example, a small percentage indicated that they would not have a personal or close interaction (eat, work, study together, hug or touch) with EVD survivors. Our findings align with another previous study that has shown how EVD-related discrimination is largely based on community fear that EVD survivors are still contagious [29]. Such discrimination has led to EVD survivors being mocked by their communities, being evicted from their homes by their property owners, losing their former jobs, and being divorced by their spouses [29–33]. Most of our study participants indicated that one’s re-acceptance into the community following an EVD infection depends largely on being able to provide a certification of treatment and recovery from an Ebola treatment centre. Similarly, previous findings demonstrated that EVD survivors were restricted from visiting public places, such as public toilets, and have experienced difficulty in trading commodities at their local market due to a community reluctance to physically handle their items or money [29, 32]. These results demonstrate that comprehensive advocacy and awareness programs are still needed, especially pertaining to preventative measures and treatment options. Ebola virus disease was historically perceived as a near certain killer in the absence of treatment; however, that is no longer the case. Supportive care, rehydration with oral or intravenous fluids, and treatment of specific symptoms are now used to improve the likelihood of survival. In addition, the provision of supportive medical care for EVD infected patients, combined with approved monoclonal antibody therapy leads to recovery for most infected individuals [7, 34].

Variability in behavioural practices was observed by district, where, for instance, residing in closer geographical proximity to affected areas resulted in residents being more alert in ensuring they protected their family members from contracting the virus. Our findings complement one prior study indicating that EVD-related fear can influence inhabitants to act with increased caution and to resort to preventive measures against the disease. These changes in human behaviour resulting from fear of the virus may lead to a decreased human to human, and human to pathogen contact rates [35].

The majority of respondents consulted during the study felt that increased personal protection could be achieved via vaccination and that the EVD vaccine was safe. The Government of Rwanda, in collaboration with the WHO and other partners, have been implementing public health measures to protect its population against EVD. Vaccination was one of the measures taken to protect health care and frontline workers in the districts at highest risk of EVD in Rwanda [6, 36]. Raising awareness of the Ebola virus, educating people about appropriate prevention measures, and vaccinating the frontline is of utmost importance for successful EVD outbreak prevention and control efforts.

### Limitations and mitigation strategy

The COVID-19 pandemic and the Government of Rwanda’s COVID-19 restrictions necessitated altering data collection methods from face-to-face interviews to virtual interviews. Although contextually expedient, conducting interviews virtually created unforeseen challenges, where roughly 50% of selected respondents refused to provide informed consent. This is probably because participants may be apprehensive about sharing sensitive information or being recorded during the virtual interviews. Consequently, the research team was required to recruit more participants to replace those who had chosen not to participate in the study.

Finally, conducting interviews online might also introduce specific methodological limitations related to information bias. For instance, the absence of face-to-face interaction may lead to misinterpretations or misunderstandings of questions, potentially influencing the responses given by participants. To address this, the research team was requested to clearly communicate with study participants.

## Conclusion

Overall, the study noted a relatively high knowledge level among the study respondents with 99.6% reporting having heard of Ebola. Such high level of awareness is a strong indication of the effectiveness of the awareness raising efforts that have been previously implemented by the Government of Rwanda. The results of this study also suggest that future communication and media efforts should be focused on knowledge dissemination in the public domain, such as available treatment options, case reporting, and the importance of positively interacting with EVD survivors. These findings, along with existing data and previous experience in communicating EVD-related information throughout the country should help guide other high-risk countries in creating an effective, evidence-based framework for controlling EVD.

### Supplementary Information

**Additional file 1.** Annexes_Study Questionnaires. 

## Data Availability

For ethical reasons, raw data generated during the current study were not made publicly available. Access to the datasets analysed may be facilitated upon reasonable request to the corresponding author.

## Abbreviations

CDC: Centers for Disease Control and Prevention
CHWs: Community Health Workers
COVID-19: Coronavirus disease 2019
DRC: Democratic Republic of the Congo
ECD: Early Childhood Development
EVD: Ebola Virus Disease
FDA: Food and Drug Administration
KAP: Knowledge, Attitude and Practices
KIIs/FGD: Key Informant Interviews / Focus Group Discussion
RBC: Rwanda Biomedical Centre
SPSS: Statistical Package for Social Science
SUDV: Sudan Ebolavirus
UNICEF: United Nations Children's Fund
WHO: World Health Organization
