# Supplementary material for: Understanding knowledge, attitudes and practices on Ebola Virus Disease: a multi-site mixed methods survey on preparedness in Rwanda
Source: BMC Public Health. 2023 Dec 5;23:2417. doi: 10.1186/s12889-023-17251-w (PMC10696806; doi:10.1186/s12889-023-17251-w)
Supplement: Supplementary file 1 — Additional file 1. Annexes_Study Questionnaires. [file 12889_2023_17251_MOESM1_ESM.docx]

## ANNEXES: Tools

### Tool 1: Household survey questionnaire

**Informed verbal consent and confidentiality of interviews**

Good morning/afternoon, Mr/Ms. My name is …………………………………… We are working on a project about knowledge, attitudes and practices around the Ebola Virus in Rwanda and in which we would like you to participate in. The interview will take about 15 minutes. All the information we obtain will remain strictly confidential and your answers and name will never be revealed. Also, you are not obliged to answer any question you do not want to, and you may stop the interview at any time. This is not to evaluate you but to check how the system functions, so please do not feel pressured to give a specific response and feel free to pass any question you do not know. Kindly answer the questions honestly, telling me about what you know, how you feel, the way you live and how you make you and the community safe from Ebola.

Do you agree to participate YES/ NO (*If yes, continue; if no, stop the interview and inform team leader or supervisor)*.

Do you agree to participate YES/ NO (*If yes, continue; if no, stop the interview and inform team leader or supervisor)*.

**NB***For this discussion please kindly note we are seeking information of Ebola and not on Corona/COVID 19**

**Date**

1. **Identification**

| 1. Name of Data Collector |  |
| --- | --- |
| 1. Province |  |
| 1. District |  |
| 1. Village/Town |  |
| 1. Sector |  |
| 1. Cell |  |
| 1. Name of nearest Hospital/ health centre |  |
| 1. Age – DOB | DOB ___/____/ _____ _____Years  Don’t know |
| 1. Gender | - - - 1. Male       2. Female |
| 1. Marital status | - - - 1. Single       2. Married       3. Divorced       4. Widowed |
| 1. Place of residence | 1. Urban 2. Rural |
| 1. Are you a household head | 1. Yes 2. No |
| 1. What is your highest level of education?   *(Circle one)* | - - - 1. None       2. Primary       3. Secondary       4. Tertiary       5. University |
| 1. What is your religion *(circle one)* | - - - 1. Christianity       2. Islam       3. Traditionalist       4. None       5. Others Specify |
| 1. What kind of work do you do? | - - - 1. Unemployed       2. Petty trader       3. Private business       4. Cross border trader       5. Farmer       6. Teacher- ECD       7. Teacher/ Lecturer       8. Driver       9. Security       10. Student /Scholar       11. Others (Specify) |

1. **Awareness Knowledge Causes Signs and Symptoms**

| 1. Have you ever heard of Ebola (EVD) | - - - 1. Yes       2. No |
| --- | --- |
| 1. What is Ebola   *(Ebola is the illness with onset of fever with no response to treatment for usual causes of fever with at least 1 of the following bloody diarrhoea, bleeding from gums, bleeding into the skin, bleeding into the eyes and urine.)* | - - - 1. Correct       2. Incorrect |
| 1. What are the signs and Symptoms of Disease? *(select all that apply)* | - - - 1. Fever       2. Headache       3. Muscle pain       4. Diarrhoea       5. Abdominal pains       6. Sore throat       7. Vomiting       8. Bleeding       9. Fatigue       10. Others (specify)____________ |
| 1. What causes Ebola? *(select all that apply)* | - - - 1. Virus       2. Bats / Monkeys / Chimpanzees.       3. God or Higher Power       4. Witchcraft       5. Other (Specify): ________       6. Don’t Know |
| 1. How does a person get Ebola?   *(select all that apply)* | - - - 1. Airborne       2. Preparing bush meat/Eating bush meat       3. Bodily fluids of infected persons       4. Mosquito bits       5. Shaking hands with infected persons       6. Sleeping (sex) with infected persons Using Utensils used by infected persons       7. Others specify____________________       8. Don’t know |
| 1. How can one prevent themselves from being infected by Ebola?   *(select all that apply)* | - - - 1. Avoid contact with infected person       2. Not eating uncooked bush meat       3. Bathing with salt and hot water       4. By avoiding mosquito bites       5. Not participating in burial rituals       6. Not attending funerals |
| 1. Have you received any training/or health education on Ebola? | - - - 1. Yes       2. No |
| 1. Who are the most trusted community leaders in case of disease outbreak |  |

1. **Risk Perceptions and Beliefs**

| 1. Have you heard of any suspected Ebola case in Rwanda or neighbouring countries over the past year? (12 months) | - - - 1. Yes       2. No   If yes, name the country(ies)________________   1. DRC 2. Uganda 3. Burundi 4. Other (Specify)_________ |
| --- | --- |
| 1. Do you think Rwanda is at at risk of Ebola outbreak? | 1. Yes 2. No 3. Not sure |
| 1. Are you personally at risk of getting Ebola? | - - - 1. Yes       2. No |
| 1. What is the level of risk to you getting infected with Ebola | - - - 1. Very high       2. High       3. Moderate       4. Low /Minimal |
| 1. Why do you think you are at risk?   *(select all that apply)* | - - - 1. I live with health care professional       2. I am a cross border trader       3. My relatives across the border had the disease       4. I work as a bush meat hunter       5. Ebola is near us       6. I am just afraid       7. Others (Specify)____________ |
| 1. Do you believe that traditional healers can treat Ebola? | 1. Yes 2. No 3. Not sure |
| 1. Do you believe that spiritual (religious) healers can treat Ebola? | 1. Yes 2. No 3. Not sure |
| 1. Would you take your relative to an Ebola treatment centre? | 1. Yes 2. No 3. Not sure  **Please explain …………………………….** |

**D, Behaviours’ and Practices**

| 1. What would you do if you suspect you/family member contacted Ebola?   *(Select one only)* | - - - 1. Do nothing.       2. Hide the sick person       3. Isolate the sick person       4. Call the hotline       5. Take patient to health centre or hospital       6. Take patient to an Ebola Treatment Centre       7. Consult a traditional healer/witchdoctor       8. Take patient to a religious healer/church       9. Others (Specify)____________ |
| --- | --- |
| 1. What action would you take to avoid you and your family getting Ebola?   *(select all that apply)* | - - - 1. Practice handwashing and good personal hygiene       2. Avoiding touching sick people or the dead       3. Not eating raw bush meat       4. Washing with salt and hot water       5. Others (Specify)____________ |
| 1. Have you changed your behaviour or practices to avoid being infected with Ebola? | 1. Yes 2. No 3. Not sure |
| 1. What is the advice you give to relatives of a person who died of Ebola? | 1. do not touch the dead body 2. call health workers/burial team 3. call funeral parlours 4. quickly bury the body secretly 5. Others (Specify)____________ |
| 1. If your family member died of suspected Ebola would you accept the MOH burial team to be wholly responsible for the preparation and the burial of the deceased member | 1. Yes 2. No 3. Not sure |
| 1. Have you been involved in any activity on Ebola and its prevention in the community***? (Before COVID-19 Lockdown)*** | 1. Yes 2. No 3. Not sure |
| 1. If yes what were the activities’ conducted   ***(Before COVID-19 Lockdown)*** | - - - 1. Training of CHW       2. Dissemination of Information to communities       3. Attending meetings       4. Distributing IEC materials |

1. **Information Communication channels and sources’**

| 1. What is your source of information on Ebola?   *(select all that apply)* | - - - 1. Radio       2. TV       3. Social Media WhatsApp/Facebook       4. Mobile Phone text messages       5. Health workers       6. Newspapers/Flyers /Posters       7. Church /religious workers       8. Traditional /Community health workers       9. Email       10. Drama       11. Newspapers |
| --- | --- |
| 1. In your own view what is the most trusted source of information? | - - - 1. Health professionals       2. Community health workers       3. NGO       4. Traditional leaders       5. Religious leader       6. Others (Specify)______________ |
| 1. What is your most preferred way of communication about EVD in case of an outbreak | - - - 1. Radio       2. TV       3. Social Media WhatsApp/Facebook       4. Mobile Phone text messages       5. Health workers       6. Newspapers/Flyers /Posters       7. Church /religious workers       8. Traditional /Community health workers |
| 1. Do you need extra information on Ebola | 1. Yes 2. No 3. Not sure |
| 1. In which areas do you need extra information |  |

1. **Attitudes towards survivors**

| 1. Would you or the community welcome back a neighbour who survived Ebola | 1. Yes 2. No 3. Not sure |
| --- | --- |
| 1. If a person had Ebola would you personally do the following |  |
| - - 1. Hugging touching | 1. Yes 2. No 3. Not sure |
| - - 1. Shake hands | 1. Yes 2. No 3. Not sure |
| - - 1. Eat in the same table | 1. Yes 2. No 3. Not sure |
| - - 1. Work or study with them | 1. Yes 2. No 3. Not sure |
| - - 1. If they are shopkeepers would you buy food from them | 1. Yes 2. No 3. Not sure |
| - - 1. If in a relationship have sex with the partner | 1. Yes 2. No 3. Not sure |
| 1. What other recommendations can you give to the Ministry on Ebola? |  |

**F Vaccination issues**

| 1. (a) Do you believe that it is important to get vaccinated to prevent Ebola?   b) **Why do you think so** | - - - 1. Yes       2. No |
| --- | --- |
|  |  |
| 1. (a) Do you believe that Vaccines against Ebola are safe 2. **Explain** | - - - 1. Yes       2. No |
|  |  |
| 1. In your opinion, what do you believe would prevent people in your community to get vaccinated against Ebola? |  |
| 1. (a) If a vaccine for Ebola is available, are you ready to get vaccinated or your family member?   **(b) Explain why?** | - - - 1. Yes       2. No |
|  |  |

**This survey is happening at a time when a country is dealing with COVID-19. We will ask a few questions on COVID-19.**

| 1. (a) Do you believe that it is important to get vaccinated to prevent Covid-19?   b) **Why do you think so** | - - - 1. Yes       2. No |
| --- | --- |
|  |  |
| 1. (a) Do you believe that Vaccines against Covid-19 are safe 2. **Explain** | - - - 1. Yes       2. No |
|  |  |
| 1. In your opinion, what do you believe would prevent people in your community to get vaccinated against Covid-19? |  |
| 1. (a) If a vaccine for Covid-19 is available, are you ready to get vaccinated or your family member?   **(b) Explain why?** | - - - 1. Yes       2. No |
| 1. Do you believe that Vaccines against COVID-19 are safe? | - - - 1. Yes       2. No |
| 1. (a) Who do you believe should get vaccinated first?   **(b) Explain why?** | - - - 1. Health Workers       2. Government Officials       3. Teachers       4. Police       5. Transport operators       6. Civilians       7. School children       8. Other (Specify)……… |
|  | ______________________ |

### Tool 2: KII Guide for Health workers

**Informed verbal consent and confidentiality of interviews**

Good morning/afternoon, Mr/Ms. My name is …………………………………… We are working on a project for UNICEF on knowledge, attitudes and practices around the Ebola in Rwanda, in which we would like you to participate in. The interview will take about 30-45 minutes. All the information we obtain will remain strictly confidential and your answers and names will never be revealed. This is not to evaluate whether the people are informed on Ebola and to check how the system will functions in an Ebola outbreak. So please do not feel pressured to give a specific response and feel free to pass any question you do not know. Kindly answer the questions honestly, telling me about what you know, how you and the people feel, the way you live and how you make you and the community can be made safe from Ebola.

**NB***For this discussion please kindly note we are seeking information of Ebola and not on Corona/COVID 19**

Do you agree to participate YES/ NO (If yes, continue; if no, stop the interview and inform team leader or supervisor).

**Date**

**Identification**

| Name | Province |
| --- | --- |
| District | Village/Town |
| 1. Sector | Cell |
| 1. Name of nearest Hospital/ health centre | DOB ____/___/________ Age_________ |
| 1. Profession _________________ |  |

1. **Awareness Knowledge Causes Signs and Symptoms**
2. Have you received any training on Ebola in the past 12 months, what information did you cover? Are there any knowledge gaps you think you still need more training on?
3. As health personal what measures are in place to prevent spread of Ebola.?
4. What other measures should be put in place to prevent spread of Ebola?
5. **Risk Perceptions and Beliefs**
6. Do you think Rwanda is at risk of Ebola outbreak? What are your reasons?
7. As a health personal are you at risk of contracting Ebola, if so, what measures are in place in the health facilities to prevent that.
8. Would you accept to work in an EVD isolation hospital/clinic? What would be your fears and concerns?
9. Do you think Rwanda is adequately prepared to deal with an outbreak of Ebola? Give reasons
10. What are the current practices by health workers to prevent EVD?
11. What role is being played health personnel in raising awareness about Ebola.
12. What are the current myths, misconceptions about EVD?
13. **Behaviours’ and Practices**
14. As health personnel what steps would you take if you had patient suspected of Ebola?
15. In the Ministry of Health, is there a safe burial team? Comment on their responsiveness
16. Have you and your health institute been involved in any awareness and prevention activities in community b4 lockdown. If yes give details
17. As Health what is your main source of information about Ebola. How comprehensive, any gaps to note?
18. In the past 12 months have there been any key messages on Ebola. What about in the past 3 months
19. In your experience do you think health personnel would discriminate persons who recovered from Ebola? Give reasons.
20. What are the gender-related cultural, social norms and traditions, , influencing health-seeking behaviours as well as family and community resilience to prevent an EVD outbreak?
21. **Information Communication channels and sources’**
22. What are the preferred media/channels of communication among community members during disease outbreaks?
23. In your view what are the most trusted source of information during disease outbreak in the community?
24. Who are the most trusted leaders in the community during disease outbreaks?
25. In the last 12 months and in the last 3 months what messages did you hear on Ebola and from which sources?
26. What challenges do community members face in accessing EVD relevant information?
27. What are the key engagement points in communities and how can they be effectively engaged with EVD-related messaging?
28. As health workers what recommendations can you give about the way Ebola information should be communicated to health workers., communities, learners and children?
29. What do you think can be done in the community to increase awareness about Ebola
30. **Recommendations**
31. Any recommendation to Government and MOH how best to inform the public and allay fears
32. **Vaccination Issues**
33. Do you believe that it is important to get vaccinated to prevent Ebola? Explain
34. Do you believe that Vaccines against Ebola are safe Explain?
35. In your opinion, what do you believe would prevent people in your community to get vaccinated against Ebola?
36. If a vaccine for Ebola is available, are you ready to get vaccinated or your family member? Explain why?

**This survey is happening at a time when a country is dealing with COVID-19. We will ask a few questions on COVID-19.**

1. Do you believe that it is important to get vaccinated to prevent COVID-19? Why do you think so?
2. Do you believe that Vaccines against Covid-19 are safe Explain?
3. In your opinion, what do you believe would prevent people in your community to get vaccinated against Covid-19?
4. If a vaccine for Covid-19 is available, are you ready to get vaccinated or your family member? Explain why?
5. Who do you think should be immunized first? Explain

### Tool 3: KII Guide for Teachers/ ECD caregivers

**Informed verbal consent and confidentiality of interviews**

Good morning/afternoon, Mr/Ms. My name is …………………………………… We are working on a project for UNICEF on knowledge, attitudes and practices around the Ebola in Rwanda, in which we would like you to participate in. The interview will take about 30-45 minutes. All the information we obtain will remain strictly confidential and your answers and names will never be revealed. This is not to evaluate whether the people are informed on Ebola and to check how the system will functions in the in an Ebola outbreak. So please do not feel pressured to give a specific response and feel free to pass any question you do not know. Kindly answer the questions honestly, telling me about what you know, how you and the people feel, the way you live and how you make you and the community can be made safe from Ebola.

**NB***For this discussion please kindly note we are seeking information of Ebola and not on Corona/COVID 19**

Do you agree to participate YES/ NO (If yes, continue; if no, stop the interview and inform team leader or supervisor).

**Date………………………………….**

**Identification**

| Name of Data Collector | Province |
| --- | --- |
| District | Village/Town |
| 1. Sector | Cell |
| 1. Name of nearest Hospital/ health centre | DOB ____/___/________ Age_________ |
| 1. Profession _________________ |  |

1. **Awareness Knowledge Causes Signs and Symptoms**
2. As Educationist (Teachers/ ECD care givers) what role are you playing/ can you play to enhance knowledge about Ebola among children.
3. What do you know about Ebola? causes, signs and symptoms,
4. How does it spread, and can this spread be prevented/ How? Any cure?
5. Knowledge of Designated Ebola treatment Centres –rationale?
6. **Risk Perceptions and Beliefs**
7. Do you think that Ebola can come into Rwanda? Are you at risk? Give your reasons.
8. What are learners’ fear and concerns? Any fears?
9. Currently are there any education programmes about risk to the children.
10. In your view how can this information be communicated.
11. What are the current myths, misconceptions about EVD?
12. **Behaviours’ and Practices**
13. What measures are currently in place or should be in place in your school environment to raise awareness about Ebola in young children
14. What would you or families do if they suspected their member to have contracted Ebola?
15. What are the current practices that you are doing to prevent EVD?
16. In your community what are some of the cultural social norms and traditions including gender that may prevent people from seeking care if suspected of Ebola?
17. Would learners/children in the school environment hug shake hands? Eat with or buy food from a person who has recovered from Ebola? Reasons/underlying causes for discrimination or non-discrimination.
18. What does the school do if a child has a family member infected with Ebola?
19. As Teachers /ECD instructors did you receive any training on Ebola? By who and Where?
20. **Information Communication channels and sources’**
21. What are the preferred media/channels of communication among community members during disease outbreaks?
22. In your view what are the most trusted source of information during disease outbreak in the community?
23. Who are the most trusted leaders in the community during disease outbreaks?
24. What are the sources of information /communication about Ebola?
25. In the last 12 months and in the last 3 months what messages did you hear on Ebola and from which sources?
26. What challenges do community members face in accessing EVD relevant information?
27. What are the existing traditional platforms of communication in the communities that can be used to communicate information about EVD?
28. **Recommendations**
29. Any recommendation to Government and MOH how best to inform the public and allay fears

.

**F. Vaccination Issues**

1. Do you believe that it is important to get vaccinated to prevent Ebola? Explain
2. Do you believe that Vaccines against Ebola are safe Explain?
3. In your opinion, what do you believe would prevent people in your community to get vaccinated against Ebola?
4. If a vaccine for Ebola is available, are you ready to get vaccinated or your family member? Explain why?

**This survey is happening at a time when a country is dealing with COVID-19. We will ask a few questions on COVID-19.**

1. Do you believe that it is important to get vaccinated to prevent COVID-19? Why do you think so?
2. Do you believe that Vaccines against Covid-19 are safe Explain?
3. In your opinion, what do you believe would prevent people in your community to get vaccinated against Covid-19?
4. If a vaccine for Covid-19 is available, are you ready to get vaccinated or your family member? Explain why?
5. Who do you think should be immunized first? Explain

### Tool 4: FGD Guide for parents/ caregivers

**Informed verbal consent and confidentiality of interviews**

Good morning/afternoon, Mr/Ms. My name is …………………………………… We are working on a project for UNICEF on knowledge, attitudes and practices around the Ebola in Rwanda, in which we would like you to participate in. The interview will take about 30-45 minutes. All the information we obtain will remain strictly confidential and your answers and names will never be revealed. This is not to evaluate whether the people are informed on Ebola and to check how the system will function in an Ebola outbreak. So please do not feel pressured to give a specific response and feel free to pass any question you do not know. Kindly answer the questions honestly, telling me about what you know, how you and the people feel, the way you live and how you make you and the community can be made safe from Ebola.

**NB***For this discussion please kindly note we are seeking information of Ebola and not on Corona/COVID 19**

Do you agree to participate YES/ NO (If yes, continue; if no, stop the interview and inform team leader or supervisor).

**Date**

**Identification**

| Name of Data Collector | Province |
| --- | --- |
| District | Village/Town |
| 1. Sector | Cell |
| 1. Name of nearest Hospital/ health centre | Participating group members |

1. **Awareness Knowledge Causes Signs and Symptoms**

A1. As parents/ caregivers what do you know about Ebola, to include cause, signs and symptoms, how it spreads, prevention and cure?

**Probe**

- What people are saying about it
- People’s Concerns

A2. Have you had any discussion with your families about Ebola? Do you have adequate information to have such discussion?

A3. Do you have any information gaps around Ebola u would like to be educated on?

1. **Risk Perceptions and Beliefs**

B1. Can we now talk about the possibility of Ebola coming into Rwanda?

**Probe**.

- Borders with countries with Ebola cases
- Cross border traders, health workers, immigration workers, transporters
- Any perceived risk to children and other dependants

B2. What are the current myths, misconceptions about EVD?

1. **Behaviours’ and Practices**

C1 What would families do if they suspected their member contracted Ebola?

**Probe.**

- Sick from Ebola/ Possibility of taking sick people to Ebola Treatment Centres
- Recovered
- Died of Ebola/role of burial teams, community acceptance to use burial teams

C2. As parents and caregivers what has been your involvement on education, awareness around Ebola before the COVID-19 lockdown?

C3. In your community/family what are some of the cultural social norms and tradition including ender that prevent people from seeking care if suspected of Ebola?

C4. Would people in the families hug shake hands. Eat with or buy food from a person who has recovered from Ebola?

C5. What are the current practices by parents and caregivers to prevent EVD?

**Probe.**

- Reasons/underlying causes for discrimination or non-discrimination.

1. **Information Communication channels and sources’**

D1. Let’s talk about information communication about Ebola with respect to trusted sources of info.

**Probe**

- Trusted sources

**E Vaccination Issues**

E1. Do you believe that it is important to get vaccinated to prevent Ebola? Explain

E2. Do you believe that Vaccines against Ebola are safe Explain?

E3. In your opinion, what do you believe would prevent people in your community to get vaccinated against Ebola?

E4. If a vaccine for Ebola is available, are you ready to get vaccinated or your family member? Explain why?

**This survey is happening at a time when a country is dealing with COVID-19. We will ask a few questions on COVID-19.**

E5 Do you believe that it is important to get vaccinated to prevent COVID-19? Why do you think so?

E6 Do you believe that Vaccines against Covid-19 are safe Explain?

E7 In your opinion, what do you believe would prevent people in your community to get vaccinated against Covid-19?

E8 If a vaccine for Covid-19 is available, are you ready to get vaccinated or your family member? Explain why?

E9 Who do you think should be immunized first? Explain

### Tool 5: FGD Guide for Community Leaders

**Informed verbal consent and confidentiality of interviews**

Good morning/afternoon, Mr/Ms. My name is …………………………………… We are working on a project for UNICEF on knowledge, attitudes and practices around the Ebola in Rwanda, in which we would like you to participate in. The interview will take about 30-45 minutes. All the information we obtain will remain strictly confidential and your answers and names will never be revealed. This is not to evaluate whether the people are informed on Ebola and to check how the system will functions in the in an Ebola outbreak. So please do not feel pressured to give a specific response and feel free to pass any question you do not know. Kindly answer the questions honestly, telling me about what you know, how you and the people feel, the way you live and how you make you and the community can be made safe from Ebola.

**NB***For this discussion please kindly note we are seeking information of Ebola and not on Corona/COVID 19**

Do you agree to participate YES/ NO (If yes, continue; if no, stop the interview and inform team leader or supervisor).

**Date**

**Identification**

| Name of Data Collector | Province |
| --- | --- |
| District | Village/Town |
| 1. Sector | Cell |
| 1. Name of nearest Hospital/ health centre | Participating group members |

1. **Awareness Knowledge Causes Signs and Symptoms**

A1. What do people know about Ebola, to include cause, signs and symptoms, how it spreads, prevention and cure?

**Probe**

- What people are saying about it
- People’s Concerns

A1. How did you, as community leaders, came to know about EVD

1. **Risk Perceptions and Beliefs**

B1. Can we now talk about the possibility of Ebola coming into Rwanda?

**Probe**.

- Borders with countries with Ebola cases
- Cross border traders, health workers, immigration workers, transporters

B2. What are the attitudes of people towards EVD?

B3. What do comm leaders and society expect, think and teach about EVD?

1. **Behaviours’ and Practices**

C1 What would families do if they suspected their member contracted Ebola?

**Probe.**

- Sick from Ebola, View on Ebola Treatment Centre
- Recovered
- Died of Ebola/role of burial teams, community acceptance to use burial teams

C2. As community leaders what has been your involvement on education, awareness around Ebola before the covid19 lockdown?

C3. In your community what are some of the cultural social norms and tradition including ender that prevent people from seeking care if suspected of Ebola?

C4. Would people in the community hug shake hands. Eat with or buy food from a person who has recovered from Ebola?

**Probe.**

- Reasons/underlying causes for discrimination or non-discrimination

C5 What are the current myths, misconceptions about EVD?

C6 What are the gender related, cultural, social norms and traditions that influence health seeking behaviours to prevent EVD outbreak?

1. **Information Communication channels and sources’**

D1 What are the preferred media/channels of communication among community members during disease outbreaks?

D2 What are the most trusted sources of communication among community members during disease outbreaks?

D3 Who are the most trusted leaders among target audiences during disease outbreaks?

D4 What is the preferred means of communicating with decision makers among community members during disease outbreaks?

D5 What challenges do community members face in accessing EVD relevant information?

D6 What are the key engagement points in communities and how can they be effectively engaged with EVD-related messaging?

D7 What are the existing traditional platforms of communication in the communities that can be used communicate about EVD?

**E Vaccination Issues**

E1. Do you believe that it is important to get vaccinated to prevent Ebola? Explain

E2. Do you believe that Vaccines against Ebola are safe Explain?

E3. In your opinion, what do you believe would prevent people in your community to get vaccinated against Ebola?

E4. If a vaccine for Ebola is available, are you ready to get vaccinated or your family member? Explain why?

**This survey is happening at a time when a country is dealing with COVID-19. We will ask a few questions on COVID-19.**

E5 Do you believe that it is important to get vaccinated to prevent COVID-19? Why do you think so?

E6 Do you believe that Vaccines against Covid-19 are safe Explain?

E7 In your opinion, what do you believe would prevent people in your community to get vaccinated against Covid-19?

E8 If a vaccine for Covid-19 is available, are you ready to get vaccinated or your family member? Explain why?

E9 Who do you think should be immunized first? Explain

### Tool 6: FGD Guide for Adolescents

**Informed verbal consent and confidentiality of interviews**

Good morning/afternoon, Mr/Ms. My name is …………………………………… We are working on a project for UNICEF on knowledge, attitudes and practices around the Ebola in Rwanda, in which we would like you to participate in. The interview will take about 30-45 minutes. All the information we obtain will remain strictly confidential and your answers and names will never be revealed. This is not to evaluate whether the people are informed on Ebola and to check how the system will functions in the in an Ebola outbreak. So please do not feel pressured to give a specific response and feel free to pass any question you do not know. Kindly answer the questions honestly, telling me about what you know, how you and the people feel, the way you live and how you make you and the community can be made safe from Ebola.

**NB***For this discussion please kindly note we are seeking information of Ebola and not on Corona/COVID 19**

Do you agree to participate YES/ NO (If yes, continue; if no, stop the interview and inform team leader or supervisor).

**Date**

**Identification**

| Name of Data Collector | Province |
| --- | --- |
| District | Village/Town |
| Sector | Cell |
| Name of nearest Hospital/ health centre | Participating group members |

1. **Awareness Knowledge Causes Signs and Symptoms**

A1. What do you know about Ebola, to include cause, signs and symptoms, how it spreads, prevention and cure?

**Probe**

- What people are saying about it
- People’s Concerns

A1. As adolescents how did you come to know about EVD?

1. **Risk Perceptions and Beliefs**

B1. Can we now talk about the possibility of Ebola coming into Rwanda?

**Probe**.

- Borders with countries with Ebola cases
- Cross border traders, health workers, immigration workers, transporters

B2. What are the attitudes of people towards EVD? (positive or negative)

B3. What are your current practices as adolescents to prevent EVD?

B4 what are the current myths, misconceptions about EVD?

1. **Behaviours’ and Practices**

C1 What would families do if they suspected their member contracted Ebola?

**Probe.**

- Sick from Ebola / Ebola Treatment Centre
- Recovered
- Died of Ebola/role of burial teams, community acceptance to use burial teams

C2. As adolescents what has been your involvement on education, awareness around Ebola before the covid19 lockdown?

C4. Would people in the community hug shake hands. Eat with or buy food from a person who has recovered from Ebola?

**Probe.**

- Reasons/underlying causes for discrimination or non-discrimination

C5 What are the current myths, misconceptions about EVD?

C6 What are the gender related, cultural, social norms and traditions that influence health seeking behaviours to prevent EVD outbreak?

1. **Information Communication channels and sources’**

D1 What are the preferred media/channels of communication among adolescents during disease outbreaks?

D2 What are the most trusted sources of communication among community members during disease outbreaks?

D3 Who are the most trusted leaders among target audiences during disease outbreaks?

D4 As youths how would like to communicate with decision makers during disease outbreak?

D5 What challenges do community members face in accessing EVD relevant information?

D6 What are the key engagement points in communities and how can they be effectively engaged with EVD-related messaging?

D7 What are the existing traditional platforms of communication in the communities that can be used communicate about EVD?

**E Vaccination Issues**

E1. Do you believe that it is important to get vaccinated to prevent Ebola? Explain

E2. Do you believe that Vaccines against Ebola are safe Explain?

E3. In your opinion, what do you believe would prevent people in your community to get vaccinated against Ebola?

E4. If a vaccine for Ebola is available, are you ready to get vaccinated or your family member? Explain why?

**This survey is happening at a time when a country is dealing with COVID-19. We will ask a few questions on COVID-19.**

E5 Do you believe that it is important to get vaccinated to prevent COVID-19? Why do you think so?

E6 Do you believe that Vaccines against Covid-19 are safe Explain?

E7 In your opinion, what do you believe would prevent people in your community to get vaccinated against Covid-19?

E8 If a vaccine for Covid-19 is available, are you ready to get vaccinated or your family member? Explain why?

E9 Who do you think should be immunized first? Explain

### Tool 2.: KII Guide for Health workers

**Informed verbal consent and confidentiality of interviews**

Good morning/afternoon, Mr/Ms. We are from Rwanda Biomedical Center /RBC. My name is …………………………………… We are working on Study to assess knowledge, attitudes and practices around the Ebola in Rwanda, in which we would like you to participate in. The interview will take about 30-45 minutes. All the information we obtain will remain strictly confidential and your answers and names will never be revealed. This is not to evaluate whether the people are informed on Ebola and to check how the system will functions in the in an Ebola outbreak. So please do not feel pressured to give a specific response and feel free to pass any question you do not know. Kindly answer the questions honestly, telling me about what you know, how you and the people feel, the way you live and how you make you and the community can be made safe from Ebola.

**NB***For this discussion please kindly note we are seeking information of Ebola and few questions on COVID 19 vaccines Acceptance**

Do you agree to participate YES/ NO (If yes, continue; if no, stop the interview and inform team leader or supervisor).

**Date**

**Identification**

| Name | Province |
| --- | --- |
| District | Village/Town |
| 1. Sector | Cell |
| 1. Name of nearest Hospital/ health centre | DOB ____/___/________ Age_________ |
| 1. Profession _________________ |  |

1. **Awareness Knowledge Causes Signs and Symptoms**
2. Have you received any training on Ebola in the past 12 months, what information did you cover? Are there any knowledge gaps you think you still need more training on?
3. As health personal what measures are in place to prevent spread of Ebola.?
4. What other measures should be put in place to prevent spread of Ebola?
5. **Risk Perceptions and Beliefs**
6. Do you think Rwanda is at risk of Ebola outbreak? What are your reasons?
7. As a health personal are you at risk of contracting Ebola, if so, what measures are in place in the health facilities to prevent that.
8. Would you accept to work in an EVD isolation hospital/clinic? What would be your fears and concerns?
9. Do you think Rwanda is adequately prepared to deal with an outbreak of Ebola? Give reasons
10. What are the current practices by health workers to prevent EVD?
11. What role is being played health personnel in raising awareness about Ebola.
12. What are the current myths, misconceptions about EVD?
13. **Behaviours’ and Practices**
14. As health personnel what steps would you take if you had patient suspected of Ebola?
15. In the Ministry of Health, is there a safe burial team? Comment on their responsiveness
16. Have you and your health institute been involved in any awareness and prevention activities in community b4 lockdown. If yes give details
17. As Health what is your main source of information about Ebola. How comprehensive, any gaps to note?
18. In the past 12 months have there been any key messages on Ebola. What about in the past 3 months
19. In your experience do you think health personnel would discriminate persons who recovered from Ebola? Give reasons.
20. What are the gender-related cultural, social norms and traditions, , influencing health-seeking behaviours as well as family and community resilience to prevent an EVD outbreak?
21. **Information Communication channels and sources’**
22. What are the preferred media/channels of communication among community members during disease outbreaks?
23. In your view what are the most trusted source of information during disease outbreak in the community?
24. Who are the most trusted leaders in the community during disease outbreaks?
25. In the last 12 months and in the last 3 months what messages did you hear on Ebola and from which sources?
26. What challenges do community members face in accessing EVD relevant information?
27. What are the key engagement points in communities and how can they be effectively engaged with EVD-related messaging?
28. As health workers what recommendations can you give about the way Ebola information should be communicated to health workers., communities, learners and children?
29. What do you think can be done in the community to increase awareness about Ebola
30. **Recommendations**
31. Any recommendation to Government and MOH how best to inform the public and allay fears
32. **Vaccination Issues**
33. Do you believe that it is important to get vaccinated to prevent Ebola? Explain
34. Do you believe that Vaccines against Ebola are safe Explain?
35. In your opinion, what do you believe would prevent people in your community to get vaccinated against Ebola?
36. If a vaccine for Ebola is available, are you ready to get vaccinated or your family member? Explain why?

**This survey is happening at a time when a country is dealing with COVID-19. We will ask a few questions on COVID-19 Vaccines**

1. Do you believe that it is important to get vaccinated to prevent COVID-19? Why do you think so?
2. Do you believe that Vaccines against Covid-19 are safe Explain?
3. In your opinion, what do you believe would prevent people in your community to get vaccinated against Covid-19?
4. If a vaccine for Covid-19 is available, are you ready to get vaccinated or your family member? Explain why?
5. Who do you think should be immunized first? Explain

### Tool 3: KII Guide for Teachers/ ECD caregivers

**Informed verbal consent and confidentiality of interviews**

Good morning/afternoon, Mr/Ms. We are from Rwanda Biomedical Center /RBC. My name is …………………………………… We are working on Study to assess knowledge, attitudes and practices around the Ebola in Rwanda, in which we would like you to participate in. The interview will take about 30-45 minutes. All the information we obtain will remain strictly confidential and your answers and names will never be revealed. This is not to evaluate whether the people are informed on Ebola and to check how the system will functions in the in an Ebola outbreak. So please do not feel pressured to give a specific response and feel free to pass any question you do not know. Kindly answer the questions honestly, telling me about what you know, how you and the people feel, the way you live and how you make you and the community can be made safe from Ebola.

**NB***For this discussion please kindly note we are seeking information of Ebola and CO few question on COVID 19 Vaccines**

Do you agree to participate YES/ NO (If yes, continue; if no, stop the interview and inform team leader or supervisor).

**Date………………………………….**

**Identification**

| Name of Data Collector | Province |
| --- | --- |
| District | Village/Town |
| 1. Sector | Cell |
| 1. Name of nearest Hospital/ health centre | DOB ____/___/________ Age_________ |
| 1. Profession _________________ |  |

1. **Awareness Knowledge Causes Signs and Symptoms**
2. As Educationist (Teachers/ ECD care givers) what role are you playing/ can you play to enhance knowledge about Ebola among children.
3. What do you know about Ebola? causes, signs and symptoms,
4. How does it spread, and can this spread be prevented/ How? Any cure?
5. Knowledge of Designated Ebola treatment Centres –rationale?
6. **Risk Perceptions and Beliefs**
7. Do you think that Ebola can come into Rwanda? Are you at risk? Give your reasons.
8. What are learners’ fear and concerns? Any fears?
9. Currently are there any education programmes about risk to the children.
10. In your view how can this information be communicated.
11. What are the current myths, misconceptions about EVD?
12. **Behaviours’ and Practices**
13. What measures are currently in place or should be in place in your school environment to raise awareness about Ebola in young children
14. What would you or families do if they suspected their member to have contracted Ebola?
15. What are the current practices that you are doing to prevent EVD?
16. In your community what are some of the cultural social norms and traditions including gender that may prevent people from seeking care if suspected of Ebola?
17. Would learners/children in the school environment hug shake hands? Eat with or buy food from a person who has recovered from Ebola? Reasons/underlying causes for discrimination or non-discrimination.
18. What does the school do if a child has a family member infected with ebola?
19. As Teachers /ECD instructors did you receive any training on Ebola? By who and Where?
20. **Information Communication channels and sources’**
21. What are the preferred media/channels of communication among community members during disease outbreaks?
22. In your view what are the most trusted source of information during disease outbreak in the community?
23. Who are the most trusted leaders in the community during disease outbreaks?
24. What are the sources of information /communication about Ebola?
25. In the last 12 months and in the last 3 months what messages did you hear on Ebola and from which sources?
26. What challenges do community members face in accessing EVD relevant information?
27. What are the existing traditional platforms of communication in the communities that can be used to communicate information about EVD?
28. **Recommendations**
29. Any recommendation to Government and MOH how best to inform the public and allay fears

.

**F. Vaccination Issues**

1. Do you believe that it is important to get vaccinated to prevent Ebola? Explain
2. Do you believe that Vaccines against Ebola are safe Explain?
3. In your opinion, what do you believe would prevent people in your community to get vaccinated against Ebola?
4. If a vaccine for Ebola is available, are you ready to get vaccinated or your family member? Explain why?

**This survey is happening at a time when a country is dealing with COVID-19. We will ask a few questions on COVID-19 vaccines**

1. Do you believe that it is important to get vaccinated to prevent COVID-19? Why do you think so?
2. Do you believe that Vaccines against Covid-19 are safe Explain?
3. In your opinion, what do you believe would prevent people in your community to get vaccinated against Covid-19?
4. If a vaccine for Covid-19 is available, are you ready to get vaccinated or your family member? Explain why?
5. Who do you think should be immunized first? Explain

### Tool 4: FGD Guide for parents/ caregivers

**Informed verbal consent and confidentiality of interviews**

Good morning/afternoon, Mr/Ms. We are from Rwanda Biomedical Center /RBC. My name is …………………………………… We are working on Study to assess knowledge, attitudes and practices around the Ebola in Rwanda, in which we would like you to participate in. The interview will take about 30-45 minutes. All the information we obtain will remain strictly confidential and your answers and names will never be revealed. This is not to evaluate whether the people are informed on Ebola and to check how the system will functions in the in an Ebola outbreak. So please do not feel pressured to give a specific response and feel free to pass any question you do not know. Kindly answer the questions honestly, telling me about what you know, how you and the people feel, the way you live and how you make you and the community can be made safe from Ebola.

**NB***For this discussion please kindly note we are seeking information of Ebola and few question on COVID 19 vaccines**

Do you agree to participate YES/ NO (If yes, continue; if no, stop the interview and inform team leader or supervisor).

**Date**

**Identification**

| Name of Data Collector | Province |
| --- | --- |
| District | Village/Town |
| 1. Sector | Cell |
| 1. Name of nearest Hospital/ health centre | Participating group members |

1. **Awareness Knowledge Causes Signs and Symptoms**

A1. As parents/ caregivers what do you know about Ebola, to include cause, signs and symptoms, how it spreads, prevention and cure?

**Probe**

- What people are saying about it
- People’s Concerns

A2. Have you had any discussion with your families about Ebola? Do you have adequate information to have such discussion?

A3. Do you have any information gaps around Ebola u would like to be educated on?

1. **Risk Perceptions and Beliefs**

B1. Can we now talk about the possibility of Ebola coming into Rwanda?

**Probe**.

- Borders with countries with Ebola cases
- Cross border traders, health workers, immigration workers, transporters
- Any perceived risk to children and other dependants

B2. What are the current myths, misconceptions about EVD?

1. **Behaviours’ and Practices**

C1 What would families do if they suspected their member contracted Ebola?

**Probe.**

- Sick from Ebola/ Possibility of taking sick people to Ebola Treatment Centres
- Recovered
- Died of Ebola/role of burial teams, community acceptance to use burial teams

C2. As parents and caregivers what has been your involvement on education, awareness around Ebola before the COVID-19 lockdown?

C3. In your community/family what are some of the cultural social norms and tradition including ender that prevent people from seeking care if suspected of Ebola?

C4. Would people in the families hug shake hands. Eat with or buy food from a person who has recovered from Ebola?

C5. What are the current practices by parents and caregivers to prevent EVD?

**Probe.**

- Reasons/underlying causes for discrimination or non-discrimination.

1. **Information Communication channels and sources’**

D1. Let’s talk about information communication about Ebola with respect to trusted sources of info.

**Probe**

- Trusted sources

**E Vaccination Issues**

E1. Do you believe that it is important to get vaccinated to prevent Ebola? Explain

E2. Do you believe that Vaccines against Ebola are safe Explain?

E3. In your opinion, what do you believe would prevent people in your community to get vaccinated against Ebola?

E4. If a vaccine for Ebola is available, are you ready to get vaccinated or your family member? Explain why?

**This survey is happening at a time when a country is dealing with COVID-19. We will ask a few questions on COVID-19 vaccines**

E5 Do you believe that it is important to get vaccinated to prevent COVID-19? Why do you think so?

E6 Do you believe that Vaccines against Covid-19 are safe Explain?

E7 In your opinion, what do you believe would prevent people in your community to get vaccinated against Covid-19?

E8 If a vaccine for Covid-19 is available, are you ready to get vaccinated or your family member? Explain why?

E9 Who do you think should be immunized first? Explain

**Tool 5: FGD/KKI Guide for Community Leaders**

**Informed verbal consent and confidentiality of interviews**

Good morning/afternoon, Mr/Ms. We are from Rwanda Biomedical Center /RBC. My name is …………………………………… We are working on Study to assess knowledge, attitudes and practices around the Ebola in Rwanda, in which we would like you to participate in. The interview will take about 30-45 minutes. All the information we obtain will remain strictly confidential and your answers and names will never be revealed. This is not to evaluate whether the people are informed on Ebola and to check how the system will functions in the in an Ebola outbreak. So please do not feel pressured to give a specific response and feel free to pass any question you do not know. Kindly answer the questions honestly, telling me about what you know, how you and the people feel, the way you live and how you make you and the community can be made safe from Ebola.

**NB***For this discussion please kindly note we are seeking information of Ebola and few questions on COVID 19 vaccines**

Do you agree to participate YES/ NO (If yes, continue; if no, stop the interview and inform team leader or supervisor).

**Date**

**Identification**

| Name of Data Collector | Province |
| --- | --- |
| District | Village/Town |
| 1. Sector | Cell |
| 1. Name of nearest Hospital/ health centre | Participating group members |

1. **Awareness Knowledge Causes Signs and Symptoms**

A1. What do people know about Ebola, to include cause, signs and symptoms, how it spreads, prevention and cure?

**Probe**

- What people are saying about it
- People’s Concerns

A1. How did you, as community leaders, came to know about EVD

1. **Risk Perceptions and Beliefs**

B1. Can we now talk about the possibility of Ebola coming into Rwanda?

**Probe**.

- Borders with countries with Ebola cases
- Cross border traders, health workers, immigration workers, transporters

B2. What are the attitudes of people towards EVD?

B3. What do comm leaders and society expect, think and teach about EVD?

1. **Behaviours’ and Practices**

C1 What would families do if they suspected their member contracted Ebola?

**Probe.**

- Sick from Ebola, View on Ebola Treatment Centre
- Recovered
- Died of Ebola/role of burial teams, community acceptance to use burial teams

C2. As community leaders what has been your involvement on education, awareness around Ebola before the covid19 lockdown?

C3. In your community what are some of the cultural social norms and tradition including ender that prevent people from seeking care if suspected of Ebola?

C4. Would people in the community hug shake hands. Eat with or buy food from a person who has recovered from Ebola?

**Probe.**

- Reasons/underlying causes for discrimination or non-discrimination

C5 What are the current myths, misconceptions about EVD?

C6 What are the gender related, cultural, social norms and traditions that influence health seeking behaviours to prevent EVD outbreak?

1. **Information Communication channels and sources’**

D1 What are the preferred media/channels of communication among community members during disease outbreaks?

D2 What are the most trusted sources of communication among community members during disease outbreaks?

D3 Who are the most trusted leaders among target audiences during disease outbreaks?

D4 What is the preferred means of communicating with decision makers among community members during disease outbreaks?

D5 What challenges do community members face in accessing EVD relevant information?

D6 What are the key engagement points in communities and how can they be effectively engaged with EVD-related messaging?

D7 What are the existing traditional platforms of communication in the communities that can be used communicate about EVD?

**E Vaccination Issues**

E1. Do you believe that it is important to get vaccinated to prevent Ebola? Explain

E2. Do you believe that Vaccines against Ebola are safe Explain?

E3. In your opinion, what do you believe would prevent people in your community to get vaccinated against Ebola?

E4. If a vaccine for Ebola is available, are you ready to get vaccinated or your family member? Explain why?

**This survey is happening at a time when a country is dealing with COVID-19. We will ask a few questions on COVID-19.**

E5 Do you believe that it is important to get vaccinated to prevent COVID-19? Why do you think so?

E6 Do you believe that Vaccines against Covid-19 are safe Explain?

E7 In your opinion, what do you believe would prevent people in your community to get vaccinated against Covid-19?

E8 If a vaccine for Covid-19 is available, are you ready to get vaccinated or your family member? Explain why?

E9 Who do you think should be immunized first? Explain

### Tool 6: FGD Guide for Adolescents

**Informed verbal consent and confidentiality of interviews**

Good morning/afternoon, Mr/Ms. We are from Rwanda Biomedical Center /RBC. My name is …………………………………… We are working on Study to assess knowledge, attitudes and practices around the Ebola in Rwanda, in which we would like you to participate in. The interview will take about 30-45 minutes. All the information we obtain will remain strictly confidential and your answers and names will never be revealed. This is not to evaluate whether the people are informed on Ebola and to check how the system will functions in the in an Ebola outbreak. So please do not feel pressured to give a specific response and feel free to pass any question you do not know. Kindly answer the questions honestly, telling me about what you know, how you and the people feel, the way you live and how you make you and the community can be made safe from Ebola.

**NB***For this discussion please kindly note we are seeking information of Ebola and few questions on COVID 19 vaccines**

Do you agree to participate YES/ NO (If yes, continue; if no, stop the interview and inform team leader or supervisor).

**Date**

**Identification**

| Name of Data Collector | Province |
| --- | --- |
| District | Village/Town |
| Sector | Cell |
| Name of nearest Hospital/ health centre | Participating group members |

1. **Awareness Knowledge Causes Signs and Symptoms**

A1. What do you know about Ebola, to include cause, signs and symptoms, how it spreads, prevention and cure?

**Probe**

- What people are saying about it
- People’s Concerns

A1. As adolescents how did you come to know about EVD?

1. **Risk Perceptions and Beliefs**

B1. Can we now talk about the possibility of Ebola coming into Rwanda?

**Probe**.

- Borders with countries with Ebola cases
- Cross border traders, health workers, immigration workers, transporters

B2. What are the attitudes of people towards EVD? (positive or negative)

B3. What are your current practices as adolescents to prevent EVD?

B4 what are the current myths, misconceptions about EVD?

1. **Behaviours’ and Practices**

C1 What would families do if they suspected their member contracted Ebola?

**Probe.**

- Sick from Ebola / Ebola Treatment Centre
- Recovered
- Died of Ebola/role of burial teams, community acceptance to use burial teams

C2. As adolescents what has been your involvement on education, awareness around Ebola before the covid19 lockdown?

C4. Would people in the community hug shake hands. Eat with or buy food from a person who has recovered from Ebola?

**Probe.**

- Reasons/underlying causes for discrimination or non-discrimination

C5 What are the current myths, misconceptions about EVD?

C6 What are the gender related, cultural, social norms and traditions that influence health seeking behaviours to prevent EVD outbreak?

1. **Information Communication channels and sources’**

D1 What are the preferred media/channels of communication among adolescents during disease outbreaks?

D2 What are the most trusted sources of communication among community members during disease outbreaks?

D3 Who are the most trusted leaders among target audiences during disease outbreaks?

D4 As youths how would like to communicate with decision makers during disease outbreak?

D5 What challenges do community members face in accessing EVD relevant information?

D6 What are the key engagement points in communities and how can they be effectively engaged with EVD-related messaging?

D7 What are the existing traditional platforms of communication in the communities that can be used communicate about EVD?

**E Vaccination Issues**

E1. Do you believe that it is important to get vaccinated to prevent Ebola? Explain

E2. Do you believe that Vaccines against Ebola are safe Explain?

E3. In your opinion, what do you believe would prevent people in your community to get vaccinated against Ebola?

E4. If a vaccine for Ebola is available, are you ready to get vaccinated or your family member? Explain why?

**This survey is happening at a time when a country is dealing with COVID-19. We will ask a few questions on COVID-19.**

E5 Do you believe that it is important to get vaccinated to prevent COVID-19? Why do you think so?

E6 Do you believe that Vaccines against Covid-19 are safe Explain?

E7 In your opinion, what do you believe would prevent people in your community to get vaccinated against Covid-19?

E8 If a vaccine for Covid-19 is available, are you ready to get vaccinated or your family member? Explain why?

E9 Who do you think should be immunized first? Explain
